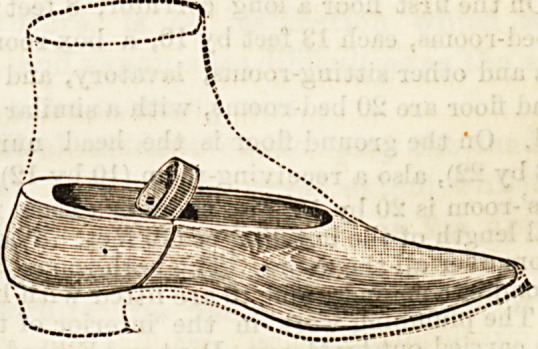# Boot Warmer and Drier

**Published:** 1893-09-23

**Authors:** 


### PRACTICAL DEPARTMENTS.

### BOOT WARMER AND DRIER.

A new invention for the warming and drying of boots and shoes has been brought out by Messrs. E. and W. Belden, Great Dover Street, S.E. It is in the form of a boot tree, hollowed out for the reception of an oblong block of metal, which is heated over a spirit lamp. Its construction is easily seen by the accompanying sketches. The bottom consists of a woollen sock which allows the boot to become quickly and thoroughly warmed. The inventors claim that the heat is retained for about three hours, and that this arrangement prevents any damage to the leather, which might arise from more direct contact with the heating apparatus.

## Figures and Tables

**Figure f1:**
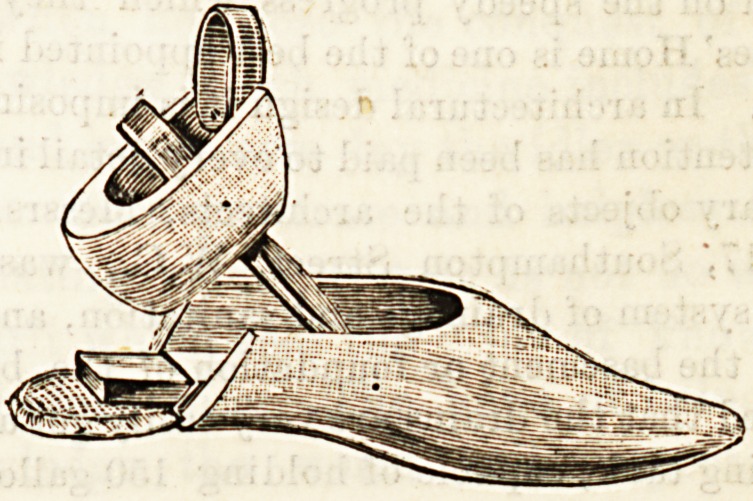


**Figure f2:**